# Application of Nanoindentation in the Characterization of a Porous Material with a Clastic Texture

**DOI:** 10.3390/ma14164579

**Published:** 2021-08-15

**Authors:** Sathwik S. Kasyap, Kostas Senetakis

**Affiliations:** Department of Architecture and Civil Engineering, City University of Hong Kong, Kowloon Tong, Hong Kong, China; ssarvadev2-c@my.cityu.edu.hk

**Keywords:** nanoindentation, hardness, modulus, porous structure, pop-in, elbowing, bowing out

## Abstract

In materials science and engineering, a significant amount of research has been carried out using indentation techniques in order to characterize the mechanical properties and microstructure of a broad range of natural and engineered materials. However, there are many unresearched or partly researched areas, such as, for example, the investigation of the shape of the indentation load–displacement curve, the associated mechanism in porous materials with clastic texture, and the influence of the texture on the constitutive behavior of the materials. In the present study, nanoindentation is employed in the analysis of the mechanical behavior of a benchmark material composed of plaster of Paris, which represents a brand of highly porous-clastic materials with a complex structure; such materials may find many applications in medicine, production industry, and energy sectors. The focus of the study is directed at the examination of the influence of the porous structure on the load–displacement response in loading and unloading phases based on nanoindentation experiments, as well as the variation with repeating the indentation in already indented locations. Events such as pop-in in the loading phase and bowing out and elbowing in the unloading phase of a given nanoindentation test are studied. Modulus, hardness, and the elastic stiffness values were additionally examined. The repeated indentation tests provided validations of various mechanisms in the loading and unloading phases of the indentation tests. The results from this study provide some fundamental insights into the interpretation of the nanoindentation behavior and the viscoelastic nature of porous-clastic materials. Some insights on the influence of indentation spacing to depth ratio were also obtained, providing scope for further studies.

## 1. Introduction

Porous materials are encountered in a large number of engineering applications, such as, for example, in petroleum/energy engineering, space exploration, biomedical, industrial, and civil engineering. Understanding of the mechanical performance of materials and the development of constitutive modeling necessitates the use of nano-to-micromechanical-based approaches as they offer insights into the relationship between bulk behavior and microstructural characteristics. Examples of this may refer to structural/cementitious materials [1,2,3,4], shale rocks [5,6,7], or ceramics [8]. Specific material properties which are of major interest in tribology engineering are Young’s modulus and (micro-)hardness, as they strongly influence the frictional response and slip displacement of interfaces [9,10]. These properties can be assessed based on indentation experiments, a type of test which offers, apart from the quantification of basic material properties, insights into the microstructure of materials, which is particularly important in assessing materials of complex textures and fabrics [11]. However, the interpretation of indentation experiments is a challenging task, particularly in correlating surface/structural characteristics of materials with their viscoelastic or creep behavior.

In the present study, the nanoindentation technique was employed to investigate the relationship between the (constitutive) stress–strain response, as observed from the indentation tests, with the structural characteristics of a highly porous material composed of plaster of Paris (PP). This material has a clastic nature and a network of pores of various sizes; thus, it can be used as a benchmark to understand the fundamental behavior of highly complex materials used in various applications, such as the construction industry [12,13] and the medical industry [14,15]. Researchers have particularly highlighted the importance of understanding the influence of micro- and macro-porous structures in gypsum plaster material on the mechanical characteristics and its fracture mechanics through various analytical models and also indentation experiments [16,17,18]. For example, the spherical indentation experiments by Devillard et al. [16] on porous gypsum highlighted that the density and porous structure primarily influence the hardness, but the size of the macropores has almost no influence on the mechanical properties. The present work emphasizes the loading history influence on the indentation load–displacement curve shapes in both loading and unloading phases, and hence unraveling the associated mechanisms.

This article is structured as follows: Section 2 provides a state-of-the-art review of the theory and formulations of equations related to the indentation technique. The interpretations from the shape of the unloading curves and also the methodology to derive basic material properties are particularly highlighted. The methodology used, including material characterization, the employed grid to perform the experiments, and the method to interpret the unloading curve from the indentation tests, is detailed in Section 3. Section 4 provides a discussion on the nanoindentation test results, highlighting the influence of previous loading history on the constitutive behavior as obtained from the stress–strain curves, the discrepancies in the data, and the influence of the microstructure of the plaster of Paris in the analysis of the test results. Limitations from the study, for example, the influence of the employed grid to perform the experiments, as well as recommendations for future research in this challenging area, are also discussed in Section 4, which is followed by an outline of the major conclusions and new contributions from the study in Section 5.

## 2. Indentation Load–Displacement Curves: Theory and Formulations

The primary mechanical characteristics derived from indentation tests are the hardness and elastic modulus of a given material. Reliable values of hardness (*H*) and elastic modulus (*E_m_*) of a given material are directly dependent on the accuracy in the estimation of the elastic contact stiffness (*S*) from the load–displacement curves. As shown in Equation (1), the reduced elastic modulus (*E_r_*) (described in Equation (2)) is directly related to the elastic stiffness and the area (*A_c_*) with a geometric factor *β* (after King [19]) used for the indenters without a body of revolution [20]. Additionally, the hardness calculation depends on the elastic stiffness, as shown in Equation (3), and the computations are performed for the load–displacement condition at maximum indentation load (*F_max_*).

(1) $$E_{r}=\frac{S}{2\beta }\sqrt{\frac{\pi }{A_{c}}}$$

(2) $$\frac{1}{E_{r}}=\frac{1-\vartheta_{m}^{2}}{E_{m}}+\frac{1-\vartheta_{I}^{2}}{E_{I}}$$

In Equation (2), *ϑ* represents the Poison’s ratio, *E* represents the elastic modulus, and the subscripts *m* and *I* represent the tested material and the indenter, respectively.

(3) $$H=\frac{F_{max}}{24.5{\left\{d_{max}-0.75\frac{F_{max}}{S}\right\}}^{2}}$$

The unloading stiffness (or elastic stiffness) is computed assuming the unloading curve as a linear function, which was upgraded by Oliver and Pharr [21], proposing a power-law fitting. It was also shown by Oliver and Pharr [21] how the power-law fitting gives constant elastic stiffness, which is independent of the fraction of the unloading curve considered for fitting, while the linear fitting of the unloading curve showed varied stiffness with different fractions of the unloading curve (from indentation tests on tungsten). Such behavior was observed not only for pure elements or minerals but also for complex geological materials such as pumice grains tested by Kasyap et al. [11], where a comparison of elastic stiffness variation with the extent of the unloading curve fitted for linear and power-law methods was discussed. They reported that the linear fitting has decreasing stiffness with increasing extent of the unloading curve (around two times), while the power-law fitting has relatively constant elastic stiffness values (for the cases with more than 10% of the unloading curve). With more complex materials, such as asphalt binders and polymers, the unloading curves of indentation tests might show negative slopes (‘nose’ shape or bowing out) due to the soft viscoelastic nature of the material where none of these methods can be used [22]. Some recommendations were proposed by Tarefdar and Faisal [22] to increase the dwell time that suppresses the viscoelastic nature of the material.

From the nanoindentation observations, mostly of brittle materials, phenomena such as pop-in (or chipping), elbowing, and pop-out were generally observed in the load–displacement curves, which define the material characteristics and their transformations during the test [23,24,25,26,27,28]. Abram et al. [29], through molecular dynamic (MD) simulations of nanoindentation on a silicon crystal, concluded that the nano-volume extrusion from the applied stress leads to pop-in behavior in the load–displacement curves. The nanoindentation experimental work by Chang and Zhang [26] clarified that the different shapes in the unloading curves from the indentation tests on silicon are merely due to volume expansion without phase transformation, and different evolution processes lead to pop-out or elbowing trends in the unloading curves. These phenomena are mostly related to the contact stresses at which such phenomenon is observed to extract some correlation between the contact stresses and phase transformation or expansion in the material. Nanoindentation experiments to evaluate the mechanical properties of porous materials (bulk ceramics sintered) were performed by Chen et al. [30]. The densification of the material below the indenter was observed to show greater influence on the indentation modulus than the hardness, and no pop-in or pop-out events were observed in the load–displacement curves.

Shales are one of the most prominently tested porous geological materials to explore their mechanical properties through nanoindentation tests [6,7,31,32,33,34]. A recent work based on nanoindentation by Wang et al. [35], on gas-bearing shale rocks particularly highlighted the development of pop-ins. The major outcomes of this work were that the pop-in events are directly related to the cracks and pores in the shale rock and the pop-in events influence the hardness values more pronouncedly compared with the elastic modulus. It was concluded in that study that higher loading rates tend to induce pop-in events in the load–displacement curves.

The contact stresses developed during the indentation test are critical to estimate the possible factors leading to irregularities in the load–displacement curves, hence the variation in the hardness and modulus values. Juliano et al. [36] used the formulations to estimate the contact stresses between the indenter and the material based on the methods (elastic recovery) developed by Oliver and Pharr [21] and Noviko et al. [37]. The contact stresses developed at every timestep (or displacement) in the indentation test can be calculated based on the area estimation from the indentation depth and the corresponding indentation load, as shown in Equations (4)–(8).

(4) $$\sigma_{j}=\frac{F_{j}}{A_{c,j}}$$

(5) $$A_{c,j}=24.5d_{c,j}^{2}+\overset{7}{\underset{i=1}{\sum }}C_{i}d_{c}^{1/2^{j}}$$

(6) $$d_{c,j}=d_{j}-d_{s,j}$$

(7) $$d_{s,j}=d_{s,max}{\left\{\frac{F_{j}}{F_{max}}\right\}}^{0.5}$$

(8) $$d_{s,max}=0.75\frac{F_{max}}{S}$$

**Figure 1:**
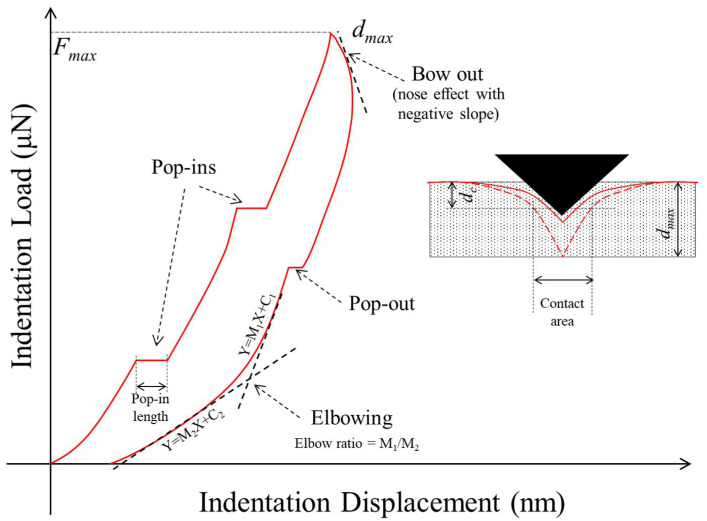
Schematic illustration explaining various events in the loading and unloading phases using a hypothetical nanoindentation load–displacement curve.

In Equations (4)–(8), the subscript *j* represents a given data point. The stress (*σ*) is a simple ratio of indentation load (*F*) to the contact area (*A_c_*). The contact area, in turn, depends on the displacement corresponding to the indenter impression (*d_c_*) and correction factors for an imperfect indenter tip (generally irrelevant at large indentation depths and omitted for the analysis in this study). The elastic displacements (*d_s_*) governing the unloading curve are dependent on the load and the unloading stiffness (*S*) based on the linear approximation of the unloading curve based on Sneddon [38]. These formulations are adopted in the present study to estimate the variation of contact stresses with indentation displacement and develop correlations with the mechanical properties of the benchmark material included in this work. A schematic illustration showing a hypothetical curve with the possible events in the loading and unloading phases, along with other parameters, is shown in Figure 1.

## 3. Materials and Methods

**Figure 2:**
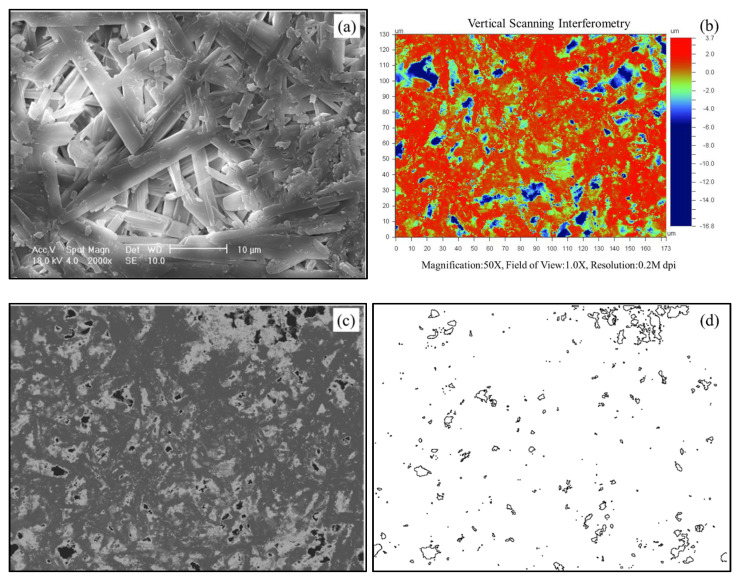
(**a**) SEM image of a PP block at 2000× magnification, (**b**) Surface profile of plaster block and associated parameters, (**c**) 8-bit image before the threshold for pore area calculation, and (**d**) boundaries of surface pores separated for area calculation using ImageJ (version 1.8.0_172).

Nanoindentation tests were performed on the plaster of Paris with the Hysitron TI 950 Nano-indenter fitted with a three-sided pyramid Berkovich single-crystal diamond indenter (elastic modulus of 1141 GPa and Poisson’s ratio of 0.07). As a procedure of calibration of the *z*-axis (loading axis) of the nanoindentation apparatus, air indents were performed before each session regulating the electrostatic force–displacement variation of the indenter. Additionally, a few trial indentation tests were performed on a standard material (e.g., aluminum) to compare the range of mechanical properties estimated with the standard values. Plaster of Paris (PP) is a gypsum plaster (CaSO_4_. 1/2H_2_O), an ultrafine white powder that crystallizes quickly when mixed with water. The hemihydrate powder was mixed with water at a water-to-plaster (*w*/*p*) ratio of 0.8 (by weight), and consecutively, the mixture was cast in molds to form small blocks feasible to be tested in the nanoindentation apparatus. The influence of the *w*/*p* ratio on the porosity of gypsum was studied by Isern and Messing [39], and it was observed that the porosity increases linearly with the *w*/*p* ratio for a given mixing time. With a *w*/*p* ratio of 0.8, the resultant PP block is a brittle-to-ductile material with a highly porous structure. Figure 2a shows scanning electron microscope (SEM) images of the PP prepared in the present study at high magnification (2000×). The conductance of the PP block surface was improved by Ag coating to acquire SEM images at high magnification and resolution. The crystallized gypsum has needle-like crystals homogeneously distributed with significant entanglement and formation of pores throughout the sample. Assuming the theoretical density of gypsum as 2310 kg/m^3^ (after [39]) and the measured bulk density to be 1100 kg/m^3^, a porosity of 0.52 was estimated for the blocks tested in the present study.

**Figure 3:**
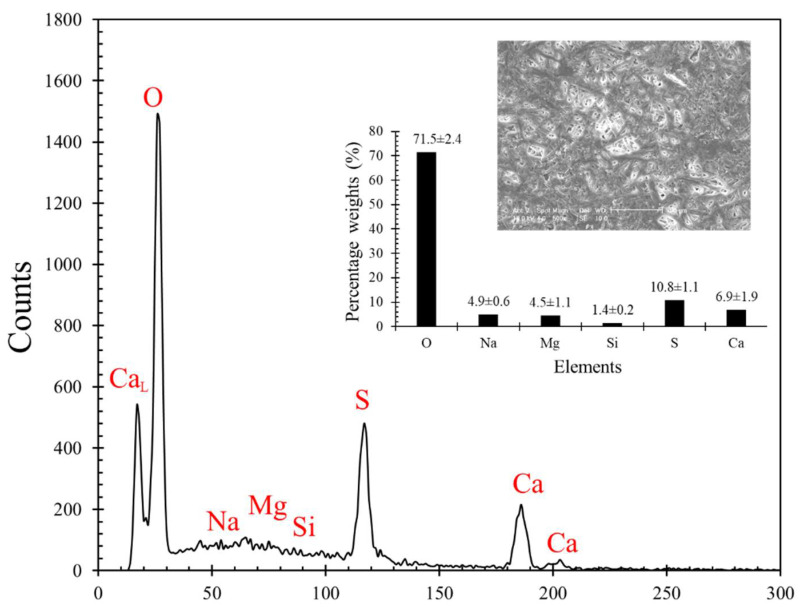
A representative EDS spectrum of PP blocks (Inset: Percentage weights of each element and a representative SEM image used for EDS analysis).

The presence of surface pores in a material highly affects the nanoindentation test results in a similar way to the body pores. An optical surface profiler (Wyko NT9300) was used to obtain the surface profiles of the plaster blocks, with an objective to observe the surface pore distribution. The vertical scan interferometry technique was adopted to obtain the surface profiles using white light. The pores in the material originating from the surface can be identified in the surface profile as large and sudden depressions compared to the rest of the surface. The surface profile of a representative plaster block is shown in Figure 2b. A set of 10 such profiles (as shown in Figure 2b), each with a scanned area of 170 μm × 130 μm were used to calculate the surface area of a pore (*A_P_*) and also the density of the pores (*D_P_*). The density of the pores is defined as the total surface area of all pores in a given surface material area of 100 μm × 100 μm. The surface area of the pores was calculated using an image processing tool (ImageJ), where the 2D images obtained from the optical surface profiler of a given scanned area were processed to threshold the 8-bit type image (Figure 2c) of the original RGB image (extract of Figure 2b). A binary image is generated after thresholding the pores, and the corresponding area of each pore can be estimated based on the pixel count (Figure 2d). For a set of 10 different samples measured, the *A_P_* values ranged between 0.11–11.52 μm^2^ (span of two orders of magnitude), and only 25% of the pores had values in the top one order of magnitude, signifying a greater number of pores being very small with an area lower than 1 μm^2^. However, these micro-scale surface pores also can greatly affect the nanoindentation results depending on the targeted indentation load and displacement. Energy-dispersive X-ray Spectroscopy (EDS) was also performed to quantify the elements present in the PP blocks (without Ag coating) tested in this study. A representative EDS spectrum is shown in Figure 3, along with the percentage weights of each element estimated from 10 different measurements. A scanned area was set as 0.05 mm^2^ for each measurement, as shown in the SEM image in Figure 3. Besides the dominant Ca, S, and O elements, some traces of impurities (Na, Mg, and Si) were also observed. The combined percentages of impurities constituted ~10.8%, signifying ~90% pure PP blocks. The scanned areas of the sample for the EDS analyses of the PP blocks were significantly larger (250 μm × 200 μm) compared to that of the nano-scale indent area of a few nm^2^. Moreover, the porous structure and the pore sizes and shapes in the plaster blocks are extremely complex (as described in Figure 2) so that they guide the variations in the mechanical properties rather than the minor mineralogical impurities.

The nanoindentation tests in the present study were conducted in a grid format with 25 indents spaced at 50 μm in both lateral directions, hence covering a 200 μm × 200 μm area on the block. The grid format is considered highly important, particularly in the indentation studies related to the chemical composition of the material to cover the surface of the material in a uniform and standard way (for example, Veytskin et al. [33]). Besides understanding the response of the material to nanoindentation loading, the objective of the present study is also to understand the variation in the material response to indentation with pre-existing damage. Hence, the second cycle of nanoindentation tests was performed on the same grid as the first indentation test. These two cycles of indentation tests were performed on two different samples to check the repeatability of the patterns in the indentation load–displacement response and hence the associated mechanical properties of the material. In the present study, power-law fitting was used with 30% of the unloading curve fitted with power function to estimate the elastic stiffness, and the cases showing the bowing out phenomenon were omitted from the analysis. A total of 100 nanoindentation tests were performed with the same maximum indentation load of 500 μN at a loading (and unloading) rate of 25 μN/s. The influence of loading rate on the indentation load–displacement response and the mechanical properties have been discussed in the literature. At different scales of loading rates ranging between 5 μN/s to 10 mN/s on many semiconductors and other minerals, one key output can be derived that the materials tend to show rapid volume changes at low loading rates, and more pronouncedly hardening behavior occurs at higher loading rates [40,41,42]. Though the present study highlights the behavior of plaster blocks at a single loading rate and single maximum indentation load, the variation in the load–displacement curves and the correlation between different phenomena in the response and mechanical properties under the influence of pre-existing indentation damage on the test results are highlighted.

## 4. Results and Discussions

### 4.1. Mechanical Properties—General Observations

**Figure 4:**
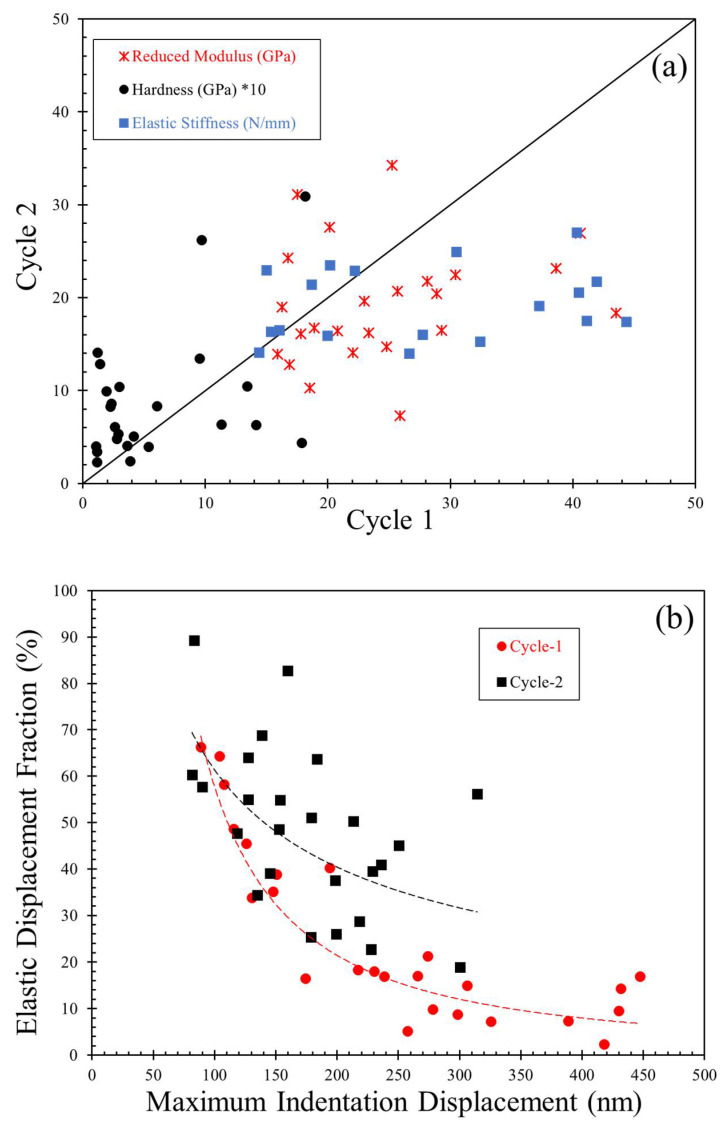
(**a**) Comparison of reduced modulus, hardness, and elastic stiffness of plaster in cycle 1 and cycle 2 of indentation. (**b**) Variation between elastic displacement fraction and maximum displacement for cycle 1 and cycle 2.

The indentation hardness and elastic modulus of plaster block were calculated based on the discussions presented in Section 2. The hardness values varied in a wide range, with the minimum and maximum values being 0.11 GPa and 1.81 GPa, respectively. The average and one standard deviation values of the indentation hardness were 0.59 ± 0.53 GPa, respectively, which correspond to a coefficient of variation of 90%. On the other hand, the elastic modulus values were less scattered compared to the hardness values, with an average and one standard deviation of 24.54 ± 7.58 GPa (coefficient of variation of 31%), implying that the influence of the porous microstructure of plaster has a greater impact on the hardness than the elastic modulus. These observations match with the interpretations discussed by Wang et al. [35] on the material properties of porous gas-bearing shales. These results correspond to cycle 1 of the nanoindentation tests on the plaster. However, performing indentation repeatedly on the exact locations provides information on the change in the mechanical properties of porous plaster under the influence of damage from the previous loading history and the possible driving mechanisms in the preceding indentation cycle. In cycle 2, the average value of hardness was measured to be 0.89 GPa (51% higher than in cycle 1) with a standard deviation of 0.69 GPa. The corresponding coefficient of variation was 77%, which is considerably lower than in cycle 1, signifying better conformity in the measured hardness values. Conversely, the elastic modulus values showed an opposite trend compared to hardness but at a smaller magnitude. The average elastic modulus value decreased in the repeated indentation tests to 19.36 GPa (about 21% lower than in cycle 1) but with a similar coefficient of variation of 31%. The decreased modulus in the cycle 2 indentation can be attributed to the reduced unloading (or elastic) stiffness. Figure 4a compares the indentation hardness, elastic modulus, and elastic stiffness values for cycle 1 and cycle 2 indentation tests for one set of data (25 tests in each cycle). A distinction in the fraction of the data points on either side of the 45° line can be observed between the hardness and modulus values. Both elastic stiffness and modulus values are statistically complying with one another in being below the 45° line (cycle 2 lower than cycle 1).

Besides the elastic stiffness, both these primary mechanical properties, hardness, and elastic modulus, are also strongly dependent on the displacements during the indentation test for the given maximum indentation load (*F_max_*). The maximum indentation displacements (*d_max_*) ranged between 88.6 nm and 447.2 nm (5 times), with the elastic fractions of the displacements (*d_e,f_*) ranging between 2.4% and 66.3% for cycle 1 of indentation. In cycle 2, the *d_max_* range reduced to 81.6–314.5 nm (3.8 times), with the *d_e,f_* values ranging between 18.9% and 89.3%. Figure 4b compares the variation of *d_e,f_* against *d_max_*, indicating a decreasing trend of elastic displacement fraction with increasing maximum indentation displacement for both indentation cycles, and the corresponding ranges of values can be observed.

The lower indentation displacements and the corresponding higher indentation hardness values in cycle 2 compared to cycle 1 suggest densification of the material resulted from the pore collapse under the indenter during the first cycle. Though the densification zones are expected to be much smaller than the elastically deformed zones [30], this case cannot be true for porous, brittle materials, as also concluded by Jauffrès et al. [43] from experimental and numerical simulations of nanoindentation on porous silica. The reduced elastic modulus values with repeated indentation related to reduced elastic stiffness are discussed in the next sections. Additionally, the reduced elastic stiffness generally corresponds to the lower elastic displacement for a linear unloading curve. The opposite behavior occurs when the unloading curve is nonlinear but with a positive elastic stiffness (i.e., no nose or bowing out phenomenon). The significant increase in the range of elastic fractions of indentation displacements in cycle 2 compared to cycle 1 implies possible abnormalities, such as elbowing or pop-in phenomena in the unloading curves of cycle 2. Given the porous, brittle nature of the tested plaster, larger indentation displacements and possible pore collapse during cycle 1 of indentation suggest a pop-in phenomenon at a greater rate of occurrence in cycle 1 than in cycle 2. The following section highlights various phenomena observed in the loading and unloading phases of the curves, their relative occurrence in two indentation cycles, and their correspondence with the mechanical properties of the plaster block tested.

### 4.2. Shapes of Loading and Unloading Curves—Related Mechanisms

#### 4.2.1. Loading Curves

Pop-ins are the most commonly observed events in the loading curve from indentation tests. During a pop-in event, the indenter displacement increases abruptly with an insignificant increase in the normal load for a small amount of displacement, and the hardening continues with further loading (Figure 1). In materials such as silica, researchers have identified that the phase transformation of the materials due to the increase in the contact stresses is the primary reason for pop-in events in the loading curves [23,25]. However, for porous materials (such as shales, plaster, or other cementitious materials), phase transformations due to temperature changes [44,45] and hence the pop-in events in such materials can be attributed to the possible dislocation networks and cracking during the indentation loading.

**Figure 5:**
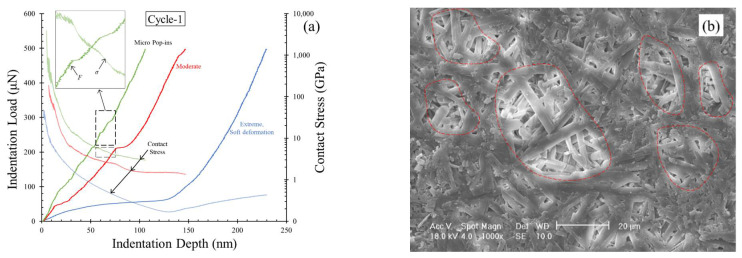
(**a**) Representative curves showing pop-in events in loading phase from the variation of indentation load and contact stress against indentation displacement for cycle 1 tests. (**b**) SEM image of plaster block highlighting weaker crystallization zones.

A set of representative indentation load–displacement curves from cycle 1 of loading on plaster tested in the present study are shown in Figure 5a. The pop-in lengths are calculated based on the absolute change in the slope of the loading curve. Three different modes of pop-in events were observed in the loading phase of the indentation tests (Figure 5a). The size (pop-in lengths) characteristics of these three modes are micro, moderate, and extreme, while the occurrence rates of these events in a given test are higher for smaller-in-length pop-ins. The micro pop-ins were the most abrupt changes in the indentation displacements, and these events were observed very frequently in a given curve, with an average of seven such events in an indentation test. However, the maximum pop-in length was 5.3 nm.

**Figure 6:**
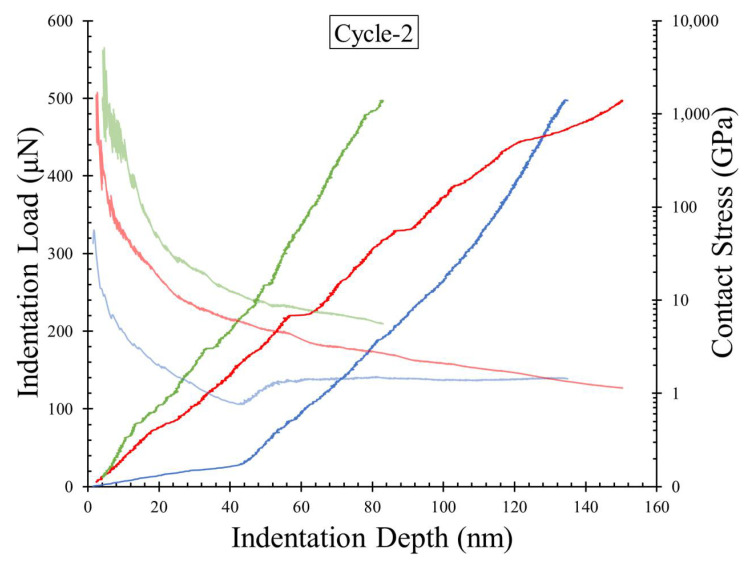
Representative curves showing pop-in events in the loading phase from the variation of indentation load and contact stress against indentation displacement for cycle 2 tests.

The second mode with moderate abruptness in the displacement had a softer behavior during the pop-in event and was less frequent in a given test with a maximum pop-in length of 15.8 nm. The extreme mode of pop-in events is associated with significantly large deformations (more than 100 nm) with an insignificant increase in the indentation load before the hardening part of the loading curve. However, these large deformations are not abrupt during the indentation test, and only one such event was observed in a given test. By attributing the pop-in events to the fracture development and the dislocations in the material during indentation, the micro and moderate modes of pop-in events can be correlated to the brittle nature of material deformation. A higher density of micropores and relatively denser interlocking of needle-shaped grains to form a continuous micro-structural fabric could lead to pop-in events with short lengths. On the other hand, regions on the plaster surface with weaker interlocks developed during the crystallization process led to soft deformations of the indenter during the loading phase. A representative set of such zones are highlighted in the SEM image produced before the indentation test (Figure 5b). These modes of pop-in events observed in indentation cycle 1 were also observed in cycle 2, but the frequency of each mode is different in the two cycles. In cycle 1, the extreme mode with soft deformation was the most frequent pop-in event, with 22 out of 50 tests (44%), while 12 tests showed micro pop-in events, and seven tests showed moderate mode pop-ins. Other tests showed a steady loading behavior with no visible abrupt changes in the displacement or potentially a micro pop-in with indistinguishably small lengths. In cycle 2, only 9 out of 50 tests (18%) showed the extreme mode with soft deformations, while the combined micro and moderate modes constituted 76% of the tests (38 out of 50). A representative set of loading curves from cycle 2 of indentation are shown in Figure 6. This phenomenon of increased sharp, micro pop-in events in cycle 2 reconfirms the densification phenomenon during cycle 1 (also observed from the decreased *d_max_* values in cycle 2), as the development of micro-cracks and dislocation during cycle 2 in the densified zones leads to more pop-in events with smaller lengths than soft deformations.

In Figure 5a and Figure 6, the contact stresses calculated from Equations (4)–(8) are also plotted, along with the indentation load–displacement curves (loading phase only). The initial stresses just after a contact is established between the indenter and the plaster block (up to 5 nm depth) are significantly high and are not plotted in these figures. As the indentation loading proceeds, a continuous decrease in the contact stresses was observed, reaching a constant stress state or continuously decelerating stress reduction before reaching the point of maximum indentation load. The inset of Figure 5a highlights the indentation load and stress variation for a certain part of the curves (dotted boxes in Figure 5a), emphasizing a sudden drop in the stresses during the pop-in events, owing to release of energy through micro-cracking and pore collapse. This phenomenon can also be observed in other modes of pop-in events. In the case of the soft deforming pop-in section, a continuous drop in the stress was observed, and in the consequent re-hardening phase of the curve, the stresses were slightly increased or had a constant value. In these re-hardening cases, no further micro pop-in events were observed, which contributes to either increased or constant stress values. Such phenomena were also observed in cycle 2 of indentation. Of the fewer tests with extreme pop-in mode with soft deformation during the loading phase in cycle 2, the stress values were observed to be constant, while the other cases with pop-in events showed a continuous decrease in the contact stresses (Figure 5a and Figure 6).

**Figure 7:**
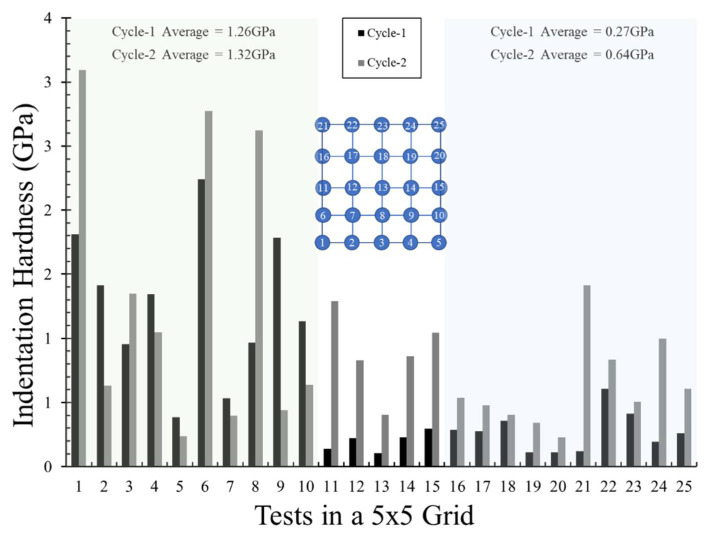
Comparison of indentation hardness between cycle 1 and cycle 2, highlighting its variation with indent location in a grid.

As discussed in Section 3, the present tests were performed in a grid of 5 × 5 with a spacing of 50 μm between each indent. An assessment of the influence of indentation spacing on the hardness and elastic modulus measurements was performed by Sudharshan Phani and Oliver [46] using nanoindentation experiments and three-dimensional finite element analyses on various materials. A critical value of indentation spacing to depth ratio was found to be equal to 10 against the previously reported value of 20 (after Samuels and Mulhearn [47]), when a Berkovich indenter is used. This work was further extended by Besharatloo and Wheeler [48] by assessing the influence of spacing on the statistical phase analysis of metal alloys. In the present study, the indentation spacing to depth ratio ranged from 110 to 610, which is significantly greater than the values proposed in the literature. However, given the extreme complexity in the mechanical characteristics of the present plaster blocks, or any other natural geological material, the minimum value of spacing to depth ratio might differ. Some observations from the current nanoindentation results suggest a more elaborated study is required to be performed on the influence of spacing, which is not the scope of the present work. As an example, Figure 7 shows the hardness values measured in 25 tests from a 5 × 5 grid with 50 μm spacing for cycle 1 and cycle 2. The inset of Figure 7 shows the scheme of the testing grid with numbers indicating the order of the indents. Isolating the first two rows of indents (tests 1 to 10) and the last two rows of indents (tests 16 to 25), the average hardness values for these two groups (highlighted in Figure 7) in cycle 1 were 1.26 GPa and 0.27 GPa (4.7 times lower), respectively. However, in cycle 2, the difference between these two groups reduced, with the last two rows of indents (tests 16 to 25) showing an average hardness value only 2 times lower than the average hardness of the first two rows. Given the substantially high indentation spacing to depth ratios, significant variation in the groups of indents is questionable. This requires a significantly large population for any statistical evaluation and is beyond the scope of the present study. However, no particular groups were observed with the shapes of loading and unloading curves, and hence it can be stated that the variation of hardness and indentation values within the grid did not affect the interpretations in the present study.

#### 4.2.2. Unloading Curves

The loading phase of the stress–displacement curves with a plateau or a decreasing trend releases the stored strain energy as the unloading phase begins. The unloading curves, as discussed in Section 2, are not always linear, and depend on the plasticity developed in the material in the vicinity of the indentation zone during the loading phase. Common phenomena or events observed in the unloading phase are pop-out (sudden decrease in displacements which is an opposite behavior to pop-in during loading), elbowing (gradual or sharp change in the unloading slope), and bowing out (negative elastic stiffness in the initial stage of unloading). All three events correspond to the relaxation of the material upon the removal of the indenter at a given unloading rate. Phase transformation of the material was attributed to the development of pop-out and elbowing events in the unloading phase [23,49], but Chang and Zhang [26] contradicted the dependence of pop-out and elbowing events to phase transformation at a given stress level. The bowing out or nose effects occur mostly in viscoelastic materials, as observed by Tarefdar and Faisal [22] for asphalt binders.

Oliver et al. [25] reviewed the occurrence of the elbowing events in the unloading curves based on their nanoindentation experimental work on crystalline germanium, which is classified as a brittle crystalline metalloid with similar physical and chemical properties as silica. It was stated that the elbowing events in the unloading phase are associated with the pop-in events in the loading phase. The location of the elbow in the unloading curve depends on the magnitude of the damage during the loading phase, and as lesser strain energy is stored during the loading, the elbow event will be faster. In the present study of nanoindentation on soft, porous plaster blocks, the test results revealed highly complex unloading responses in both cycle 1 and cycle 2. Three categories of unloading curves are observed (elbowing, nose effect, or eventless) in both indentation cycles, but the proportions of each category are different between cycle 1 and cycle 2.

**Figure 8:**
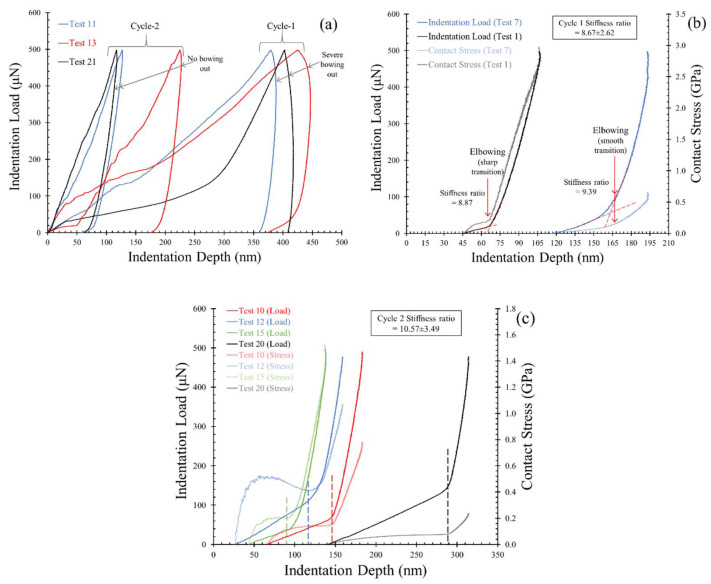
Representative curves showing events in the unloading curves (**a**) bowing out effects in cycle 1 and cycle 2, (**b**) elbowing in cycle 1, and (**c**) elbowing in cycle 2.

Nose effect (or bowing out) was observed in a limited number of tests (6 out of 50 tests: 12%) and only in cycle 1 of loading (Figure 8a). These tests were discarded from the estimation of hardness and modulus values. As no tests with significant bowing out shape were observed in cycle 2 (very minor bowing out in a few cases) of the indentation tests, this justifies the termination of viscoelastic effects in the plaster block after the first cycle of indentation (with 500 μN of load). Additionally, the few cases that showed bowing out had undergone significant soft deformations (the top 30% displacements of the group) during the loading phase. As discussed in Section 4.2.1, cycle 1 of the indentation tests primarily densified the material in the indentation zone during the loading phase, and the cases with viscoelastic bowing out are significantly recovered in cycle 2. Three such examples are shown in Figure 8a, where the extensive bowing out (nose shape) curves in cycle 1 were recovered in cycle 2 (paired in colors). Tarefdar and Faisal [22] stated that the bowing out effect can be eliminated by increasing the dwell time allowed at the maximum normal load (to dissipate viscoelasticity) for asphalt binders. The present nanoindentation tests without dwell time showed that the viscoelasticity in plaster is not a recurring effect, even with indentation loads as low as 500 μN.

From Section 4.2.1, it was understood that the micro pop-in events in cycle 2 of the indentation tests (loading phase) were much more frequent than in cycle 1, owing to the densification of the indentation zones. Correspondingly, the elbowing events in the unloading phase occurred, predominantly, in cycle 2, which is in accordance with the observations reported by Oliver et al. [25]. In cycle 1 of the unloading tests, the elbowing events were observed only in 8 out of 50 tests, and only two cases showed a sharp change in stiffness, while others showed a gradual change. Examples of load–displacement curves showing both such elbow events are shown in Figure 8b, along with their corresponding contact stress variations with indentation displacement. The points of elbow events are indicated with arrows in Figure 8b, and the ratio of the slopes of the straight lines fitting the unloading curves before and after the elbowing is 8.67 ± 2.62 (minimum of 4.58 and maximum of 11.85). The location of these elbowing events also signifies the governing mechanisms in the loading phase. If the elbow event initiates nearer to the *F_max_* (i.e., soon after the start of unloading phase), then the compressive stresses generated during the loading phase would be very limited. Delayed elbow event in unloading indicates that the compressive stresses are gradually released, and the change in the slope (elbow) occurs due to the sudden upliftment of the material associated with the crack opening. In the present study, the indentation tests in cycle 1 tests showed elbow events at the end of the unloading phase and not before the lower quarter (125 μN) during unloading.

In cycle 2, 90% of the tests (45 out of 50 tests) showed elbowing events in their unloading curves; the elbowing events also occurred earlier in the unloading phase compared with cycle 1 (representative curves are shown in Figure 8c). These early elbowing events signify that the elastic strain energy was released faster, and the opening of fractures was possible. Compared to the cycle 1 elbowing events, the change of the slope is sharp, with a ratio of 10.57 ± 3.49 (minimum of 3.76 and maximum of 17.64). The ratios are higher in cycle 2, and this strong change in slope during the elbow events suggests greater fractures around the indentation zone, which open during unloading, and the significant upliftment of the indenter by the nano-debris created in the loading phase.

## 5. Conclusions

Nanoindentation tests were conducted on blocks of plaster of Paris (PP) in the present study, employing a grid format with 25 indents spaced at 50 μm in both lateral directions, covering, in this way, a 200 μm × 200 μm area on the block. Emphasis was placed on understanding the influence of previous loading history on the stress–strain response of the material, which loading history causes (due to pre-application of indentation) damage on the surface of the PP block, thus influencing the consecutive nanoindentation results. For this purpose, two loading–unloading cycles were applied for each contact zone of the predefined grid. The major conclusions from the study are summarized as follows:

1. Based on the resultant coefficient of variation from cycle 1 of the indentation tests, it was revealed that the porous microstructure of the PP block influenced more pronouncedly the hardness values compared with the modulus values (i.e., hardness exhibited a much higher coefficient of variation in cycle 1 compared with modulus). However, better conformity of the hardness values was shown in cycle 2 with significantly reduced coefficient of variation values;
2. The test data showed lower indentation displacements and correspondingly higher indentation hardness values in cycle 2 tests compared to cycle 1, which suggests densification of the material resulting from the pore collapse under the indenter during the first cycle. It was interpreted, due to the highly porous and brittle nature of the PP blocks, that cycle 1 of indentation led to a pop-in phenomenon and significant soft deformations occurred in the loading phase, causing possible pore collapse;
3. Regions of the PP blocks with weaker interlocks led to a softer and ductile response, whereas pop-in behavior was majorly of brittle nature and occurred in regions with the denser interlocking of the microparticles;
4. Major observed modes of behavior of the unloading phase could be classified as (i) elbowing (gradual or sharp change in the unloading slope), and (ii) bowing out (negative elastic stiffness in the initial stage of unloading) and they are related with the relaxation of the material upon unloading. Additionally, significant increases in pop-in events in the loading phase and elbowing events in the unloading phase of cycle 2 tests reconfirmed the densifications of the indent zones during cycle 1 test;
5. Unlike most minerals whose indentation tests revealed their dominant material behavior to be ductile, brittle, or viscoelastic, the present material (PP), which has applications ranging from the construction industry to medicine, showed a highly complex behavior with a mix of ductile deformations, brittle breakage, and often viscoelastic deformations;
6. Some indications of indentation spacing to depth ratio influence on the mechanical properties were observed, despite the ratio being greater than 100, suggesting a need for further studies on the aspect of indentation spacing to depth ratio influence on highly porous and clastic materials. Additionally, given the highly complex porous structure of the PP blocks, a quantitative correlation between the pore attributes and indentation tests using grid-based tests can provide a much deeper understanding of their deformation mechanisms.

## Data Availability

Data are available by the corresponding author after reasonable request.
